# Organic barn dust inhibits surfactant protein D production through protein kinase-c alpha dependent increase of GPR116

**DOI:** 10.1371/journal.pone.0208597

**Published:** 2018-12-13

**Authors:** David Schneberger, Jane M. DeVasure, Shelley A. Kirychuk, Todd A. Wyatt

**Affiliations:** 1 Department of Internal Medicine, Pulmonary, Critical Care, Sleep & Allergy Division, University of Nebraska Medical Center, Omaha, Nebraska, United States of America; 2 Canadian Centre for Health and Safety in Agriculture, University of Saskatchewan, Saskatoon, Saskatchewan, Canada; 3 Department of Environmental, Agricultural, & Occupational Health, University of Nebraska Medical Center, Omaha, Nebraska, United States of America; 4 Department of Veterans Affairs, Research Service, VA Nebraska-Western Iowa Health Care System, Omaha, Nebraska, United States of America; Auburn University College of Veterinary Medicine, UNITED STATES

## Abstract

Prolonged exposure to organic barn dusts can lead to chronic inflammation and a broad range of lung problems over time, mediated by innate immune mechanisms. The immune surfactant or collectin surfactant protein D (SP-D) is a crucial multifunctional innate immune receptor. Little work to date has examined the effect of such collectins in response to organic dusts. We provide evidence here that agricultural organic dusts can inhibit mRNA and protein expression of SP-D in a human alveolar epithelial cell line, and an in vivo mouse model. This inhibition was not a result of lipopolysaccharide (LPS) or peptidoglycans, the two most commonly cited immune active components of these dusts. We further show that inhibition of the signaling molecule protein kinase C alpha (PKCα) can reverse this inhibition implicating it as a mechanism of SP-D inhibition. Examination of the SP-D regulatory receptor GPR116 showed that its mRNA expression was increased in response to dust and inhibited by blocking PKCα, implicating it as a means of inhibiting SP-D in the lungs in response to organic dusts. This reduction shows that organic barn dust can reduce lung SP-D, thus leaving workers potentially at risk for a host of pathogens.

## Introduction

Organic dusts such as those found in concentrated animal feeding operations (CAFOs) are known to cause chronic inflammation in the lungs of workers, as well as predispose them to a number of health problems such as COPD, asthma, chronic bronchitis and a general decrease in lung function over the course of their careers [1–5]. These responses have been mainly attributed to innate immune responses to key dust components such as peptidoglycans and lipopolysaccharide acting through toll-like receptors (TLR)2 and TLR4 respectively [6,7], though other ligands and receptors are implicated as having some effect as well [8,9]. Still, these studies usually do not account for the full response that is seen to such dusts, implicating other innate immune pathways and ligands.

Surfactant protein D (SP-D) is one of four protein surfactants produced by the lungs [10]. Two of these surfactants, SP-D and SP-A, sometimes referred to as collectins, appear to function primarily as innate immune molecules. Both are extremely versatile, binding a broad range of pathogens [11]. Elimination of these pathogens is accomplished by agglutination, binding causing inactivation by blocking of receptors, and opsonization [11]. Another key function of these proteins is the regulation of immune responses. This can be accomplished through binding and blocking immune receptors such as TLR4 [12], but also through the binding of either SIRP-1α or calreticulin/CD91 [13]. This last binding is dependent upon binding targets or nitric oxide (NO) modification of SP-D or SP-A [13,14]. Binding to calreticulin/CD91 by modified collectins results in phosphorylation of p38 mitogen-activated kinase (p38) causing inflammation. However, binding to SIRP-1α leads to inhibition of the same p38 [13]. Thus, SP-A and SP-D may be critical to a number of responses, and the concentration of each has implications for neutralization of pathogens and driving pro- versus anti-inflammatory signaling.

Because little information on the effects of organic dust on surfactant exists, we examined the effects of one surfactant, SP-D, on an alveolar epithelial cell line. We show here that settled organic dusts from several agricultural operations (pig barns, grain elevator) caused significant reductions in protein or mRNA expression of SP-D. These changes did not rely on endotoxin and peptidoglycan and were heat labile. The signaling enzyme protein kinase C alpha (PKCα), which is critical to the dust inflammatory cascade [15–17], was necessary for this SP-D mRNA reduction, but not PKC epsilon. Examination of the SP-D regulatory receptor GPR116 showed that it was increased by the same dust exposure, and this increase was also PKCα-dependent.

## Materials and methods

### Dust samples, extracts, and treatments

Barn dust extracts (HDE) were prepared from settled dust samples collected from the University of Saskatchewan Prairie Swine Center and from several swine facilities in Nebraska. The samples from these facilities have been characterized for bacterial composition as well as protein, endotoxin, and muramic acid [18,19]. Dust extracts were prepared as described previously [15]. Briefly, dust was mixed with Hank’s balanced salts solution (HBSS; pH 7.4; Gibco/Life Technologies/Thermofisher, Grand Island, NY) without calcium at a concentration of 1 g dust per 10 ml HBSS. This mixture was mixed for 3 min, set aside for 1 hr at room temperature, and centrifuged twice at 5000 x g for 5 minutes each time to remove large particulates. This solution was then filter sterilized (0.22 μm) for a final concentration of approximately 0.105 g/ml dust. No stability problems are noted in such extracts for at least a year or more after storage at -20°C. Extracts were used at a concentration of 5% v/v or about 0.005 g/ml dust. For inactivated dust, dust samples were baked for 24 hours at 120°C similar to methods used to remove endotoxin [20] activity prior to making some extracts. LPS used was from *Escherichia coli* O55:B5 (Sigma, St. Louis, MO) and PGN was from *Staphylococcus aureus* (Sigma, St. Louis, MO). Collectin was isolated from pulmonary alveolar proteinosis lung lavages and purified as described previously [21].

### Cell culture, treatments and inhibitors

A human alveolar epithelial cell line, A549, was purchased from American Type Culture Collection (ATCC, Manassas, VA), cultured and grown in tissue culture flasks at 37°C and 5% CO_2_ in DMEM (Gibco), supplemented with 10% fetal bovine serum (FBS) containing penicillin and streptomycin (Gibco). Cell monolayers were harvested by treatment with TrypLE Select (Gibco) for 8 minutes at 37°C. Media was added at end of incubation and centrifuged at 1000 x g for 10 minutes to wash cells and replace media. Cells were counted using a hemocytometer. Cells were cultured in either 6-well plates (0.5 x 10^6^ cells) for protein samples, or 12-well plates (0.2 x 10^6^ cells) for mRNA and allowed to attach overnight before media was replaced, cells washed with phosphate buffered saline (PBS), and media replaced with treatments in serum free media. Media was saved from the 6-well plates for ELISA. A THP-1 cell line (ATCC) was cultured and grown in tissue culture flasks at 37°C and 5% CO_2_ in RPMI (Gibco), supplemented with 10% fetal bovine serum (FBS) containing penicillin and streptomycin (Gibco). Cells were centrifuged at 1000 x g for 10 minutes to wash cells and replace media. THP-1 cells were cultured at 1.0 x 10^6^ cells/well (6-well) with 5 ng/ml phorbol myristate acetate (PMA) and allowed to bind to wells for two days before being washed with PBS and media replaced with serum-free RPMI just prior to treatment.

A549 cells were treated for 24 hr with either media alone, 5% v/v HDE extract, or 20 μg/mL SP-D. Cells treated with PKCα inhibitor Gö6976 or PKCɛ inhibitor Ro 31–8220 (Tocris, Minneapolis, MN) were given 1 mM 1 hr before treatment with media or HDE for 24 hr.

### Animals

All procedures were approved by the University of Saskatchewan Committee on Animal Care Assurance and all experiments conducted according to guidelines of the Canadian Council on Animal Care. Female, 8–10-week-old C57BL/6 mice (Charles River, Quebec) were group-housed for several weeks, and fed with commercial rodent chow and water *ad libitum*. Before exposure, an acclimatization period of 2 hr/day for 1 week to whole body plethysmography exposure chambers (DSI, Wilmington, NC) was done. Animals were assigned randomly to saline or HDE extract treatment groups. Mice were treated by single nasal instillation of 20 μl of each treatment under isoflurane anesthesia to the nares of the nose and allowed to inhale the treatment. Mice were monitored for a total of 6 hr in a plethysmography chamber before sacrifice, with no significant changes in lung function observed. All treatments were split over 2 separate days in the same week. No signs of stress or weight loss in mice were observed throughout the course of the study. Sacrifice was done via cervical dislocation of mice under isoflurane anesthesia.

### Bronchoalveolar lavage (BAL)

Lungs were lavaged as described previously [8]. Lungs were washed 3 times with 1 ml sterile saline each time. BAL fluid was centrifuged at 1750 x g for 10 minutes to remove cells, and supernates stored at -80°C. Cells from lavage were quantified using a hemocytometer and centrifuged onto glass slides which were subsequently stained using the Protocol Hema 3 kit (Thermo Fisher, Pittsburgh, PA) and cell types identified by microscopic examination.

### ELISA

Quantification of IL-6, KC and SP-D in BAL fluid was done by enzyme-linked immunosorbent assay (ELISA) kits to these proteins (R&D Systems, Minneapolis, MN) as well as IL-6 and IL-8 in cell cultures (Invitrogen/ThermoFisher, Pittsburgh, PA) according to manufacturer’s instructions. SP-A ELISA was from Cloud-Clone (Cloud-Clone, Houston, TX). Plates were read on a BioTek Synergy HT plate reader (BioTek, Winooski, VT).

### PKC activity quantification

PKCα and PKCε activity were tested via a radiolabeled ATP assay to confirm activity of PKC inhibitors. Method used was described previously (Poole et al 2007). Briefly, A549 cells were pre-incubated for 60 minutes with media, Gö6976, or Ro 31–8220 followed by a 5 hour exposure with HDE at the previously mentioned concentration. Cells were fractionated and cell extracts tested. For PKCα, cell extract was added to 900μmol/L PKC substrate peptide (Bachem, Torrence, CA), 12mmol/L calcium acetate, 8 μmol/L phosphatidyl-L-serine, 24 μg/ml phorbol 12-myristate 12-acetate, 30 mmol/L dithiothreitol, 150 μmol/L ATP, 24mmol/L magnesium acetate, and 10μCi/ml [γ-^32^P]ATP (ICN, Biomedicals, Costa Mesa, CA) in a TRIS-hydrochloric acid buffer (pH7.5) for PKCα. For PKCε, substitution of a specific PKCε substrate peptide (Calbiochem, San Diego, CA) in absence of calcium in the reaction mix was made. Kinase activity was counted in a nonaqueous scintillant in relation to total cell protein assayed and calculated in picomoles of incorporated phosphate per minute per milligram.

### RNA purification and RT-PCR analysis

Lung tissue was homogenized using Zirconia beads (BioSpec, Bartlesville, OK) with RLT lysis buffer (Qiagen, Chatsworth CA) in a Mini-Beadbeater-24 homogenizer (Biospec) for two, 1-minute rounds, with samples cooled in ice between rounds. Completion of mRNA isolation was done using a Qiagen spin miniprep plus kit (Qiagen) according to manufacturer’s instructions. Final mRNA samples were quantified using a BioTek Synergy HT plate reader (BioTek).

cDNA synthesis was accomplished by using the Taqman reverse transcription kit (Applied Biosystems, Branchburg, NJ) with 500 ng of template mRNA using the manufacturer’s instructions. Samples were incubated at 25°C for 10 min, then 48°C for 30 min, and 95°C for 5 min in a thermocycler (MJ Mini; Bio-Rad, Hercules, CA).

RT-PCR was performed with probes for human SP-D (Life Technologies, Hs00358340_m1), mouse SP-D (Life Technologies, Mm486060_m1), human GPR116 (Life Technologies, Hs00234713_m1) and mouse GPR116 (Life Technologies, Mm1269030_m1). Ribosomal RNA was used as an endogenous control. PCR was completed using an ABI PRISM 7500 Sequence Detection System (Applied Biosystems) and reactions were done for 2 min at 50°C, 10 min at 95°C, followed by 40 cycles at 95°C for 15 s and 60°C for 1 min. All reactions were done in duplicate. For relative comparison of targets to ribosomal RNA endogenous control, we analyzed cycle threshold (CT) value of real-time PCR data with the ΔΔCt method.

### Data analysis and statistics

Data was analyzed using GraphPad Prism (GraphPad Software, San Diego, CA). Error bars denote mean +/- SEM. Statistical significance was determined using ANOVA with follow-up Student’s t-test with *p* ≤ 0.05 being considered significant.

## Results

### SP-D is reduced in response to organic dusts in vitro and in a mouse model

**Fig 1:**
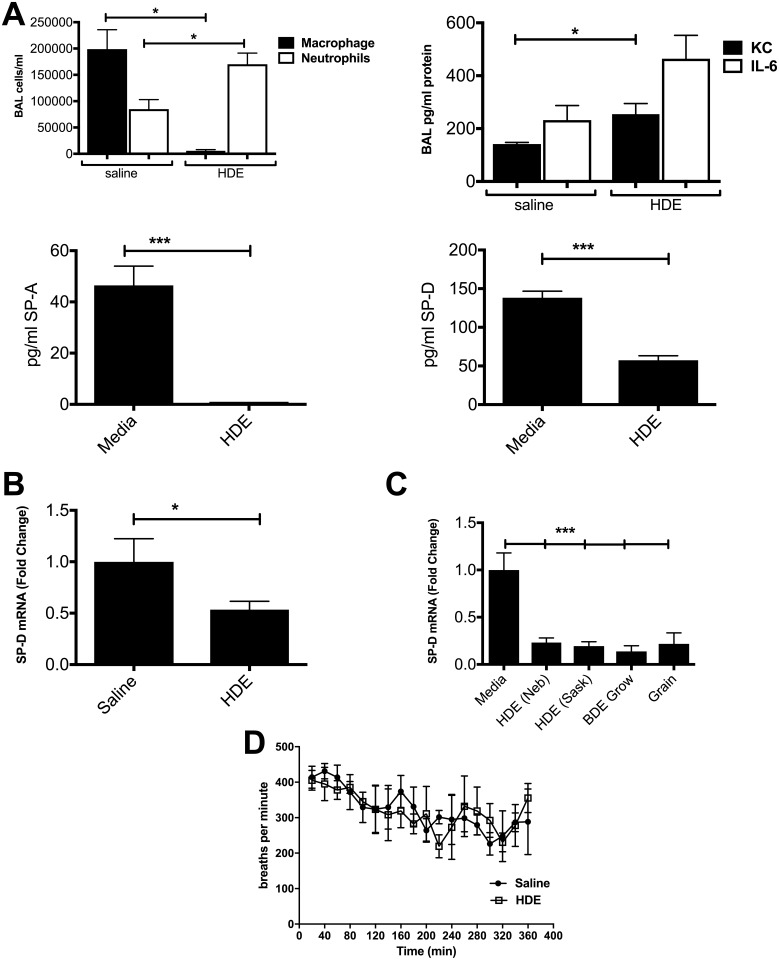
Reduced expression of SP-D to HDE extracts in an *in vivo* mouse model system. Mice exposed to HDE show slight increase in IL-6 and IL-8 and an influx of neutrophils (a). SP-A and SP-D protein in BAL (b) as well as SP-D mRNA (b) in lung tissue showed significant decreases with dust treatment. A549 cell line (c) treated for 24 hr with sterile barn hog barn dust (HDE) or grain dust extracts showed the same response in SP-D mRNA. Plethysmographic measurement of mice during exposure showed no significant change in breathing (PenH) (d). Bar graphs represent averages with error bars denoting SEM (N = 4 mice/group). Statistical significance denoted by asterisks (*p < 0.05, ***p < 0.001) as compared to respective media or saline treatment group.

Settled dust sample extracts from a hog barn in Nebraska (HDE) were tested by nasal instillation with 12.5% HDE into C57BL/6 mice. Response to HDE was consistent with previously reported results in that there was increased expression of IL-6 and KC, as well as an increase in BAL cells due in large part to an influx of neutrophils (Fig 1a). Plethysmography showed no significant changes in measured enhanced pause (Penh) readings over the exposure time (results not shown). Tests for SP-A and SP-D protein (Fig 1a) showed clear reductions with HDE treatment. Protein levels in cell cultures were typically at the lower end of ELISA detection limits, so mRNA was used as the primary indicator of SP-D levels. Choosing to follow-up on SP-D results, mRNA for SP-D from mouse lungs showed a decrease with HDE treatment similar to what was seen with proteins (Fig 1b).

Treatment of a human lung alveolar epithelial cell line (A549), with 5% v/v dust extracts for 24-hours (Fig 1c) showed that dusts from a grain elevator and two hog barns in Saskatchewan (HDE (Sask) and HDE (GRO)) were also able to significantly reduce SP-D mRNA expression, showing that this effect was not specific to a single barn or type of facility. This shows the pattern of SP-D inhibition by dust is similar in whole animal models and that mRNA expression matches SP-D protein expression in this model. Nebraska hog dust extract (HDE) samples were used for the remaining trials.

### In vitro reduction in SP-D is not affected by peptidoglycan or LPS

**Fig 2:**
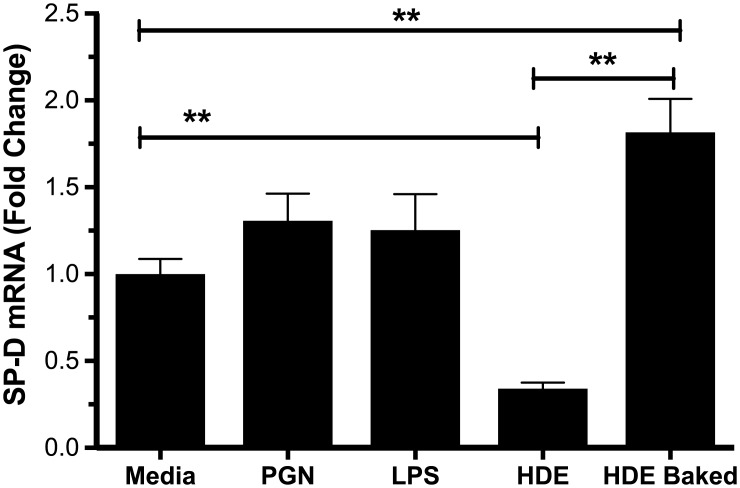
SP-D is not inhibited by LPS or peptidoglycan, but by a heat labile fraction of barn dust. A549 cells were exposed to HDE, LPS (100 EU/ml), peptidoglycan (10 μg/ml) or baked dust extract (5% v/v). Bar graphs represent averages with error bars denoting SEM (Cell cultures, N = 3 samples/group, repeated on three separate days). mRNA is given as fold change above media control levels. Statistical significance denoted by asterisks (**p < 0.01) as compared to respective media treatment group.

As endotoxin and peptidoglycan are commonly believed to be responsible for most of the immune effects seen in response to organic dusts, we treated A549 cells with LPS or peptidoglycan for 24 hours and measured surfactant. Neither treatment had any effect on SP-D mRNA (Fig 2). Heat inactivation of the dust sample on the other hand was able to abrogate SP-D inhibition, and actually caused a significant increase over control. This suggests that other components beyond LPS and peptidoglycan can have an important impact on lung innate immunity, particularly with regards to SP-D.

### SP-D reduces IL-8 response to barn dust

**Fig 3:**
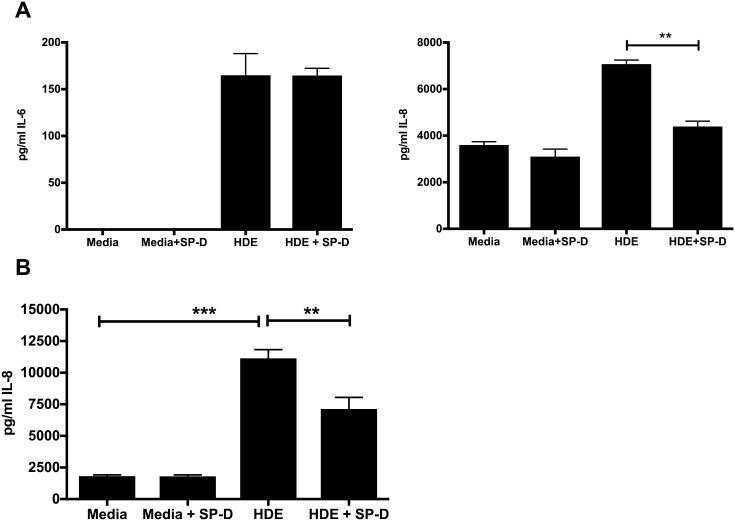
**IL-6 and IL-8 response to barn dust is inhibited by SP-D. ELISA of A549 cells** (a) or THP-1 cells (IL8 alone) (b) treated with SP-D (20 ng/ml) 1 hr prior to HDE exposure show a clear reduction in IL-8 expression 24 hr after HDE exposure. Bar graphs represent averages with error bars denoting SEM (Cell cultures, N = 3 samples/group, repeated on three separate days). mRNA is given as fold change above media control levels. Statistical significance denoted by asterisks (**p < 0.01) as compared to respective media treatment group.

We next asked if SP-D levels were relevant to inflammatory chemokine responses to barn dust. We treated A549 cells with 20 mM purified human SP-D 1 hour prior to treatment with 5% v/v barn dust, and looked for expression of IL-6 and IL-8, two commonly tested for lung inflammatory cytokines. We show that SP-D can significantly inhibit IL-8 production to dust, but appeared to have no effect on IL-6 (Fig 3a). While the levels we use are high compared to what we see in mouse lavage, it is clear that SP-D has a significant impact on the inflammatory cytokine production of A549 cells to dust exposure. A similar test using PMA-differentiated THP-1 cells showed that these cells also reduced their IL-8 expression to barn dust if pre-treated with SP-D (Fig 3b), though IL-6 was not tested given its earlier lack of change to SP-D in A549 cells.

### Protein kinase C alpha is necessary for SP-D mRNA reduction

**Fig 4:**
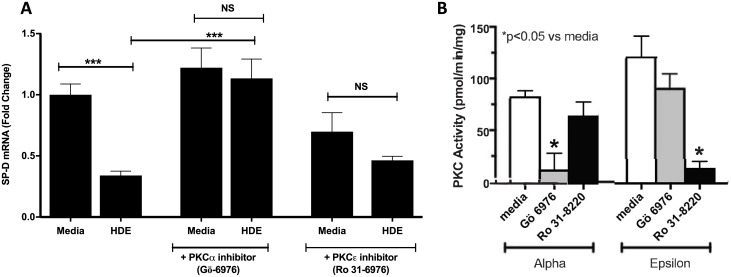
**PKCα, but not PKCɛ, is required for HDE-induced SP-D reduction** (a). Inhibitors Gö6976 (PKCα) or Ro 31–8220 (PKCɛ were given 1 hr prior to treatment of cells with HDE for 24 hr. Bar graphs represent averages mRNA fold change above media control with error bars denoting SEM (Cell cultures, N = 3 samples/group, repeated on three separate days). (b) Inhibitor specificity of Gö6976 and Ro 31–8220 in PKCα and PKCɛ activity assays. Values given are in pmol/min/mg vs. media control. Statistical significance denoted by asterisks (*p < 0.05, ***p < 0.001) as compared to respective media treatment group.

As we have previously shown that barn dust causes a cascade of PKC activation [16], which is vital to inflammatory cytokine production, we wondered if PKCα or PKCɛ played any role in SP-D mRNA regulation. Using previously characterized PKC inhibitors in the HDE signal pathway [15–17], we showed that inhibition of PKCα, but not PKCɛ, reverses SP-D mRNA reduction, returning levels to near that of control (Fig 4a). Thus, PKCα is necessary for the SP-D reduction in response to organic dust. While treatment with Ro 31–8220 also rendered SP-D reduction by HDE no longer significant, this looks to be due to a mild decrease in base expression of SP-D and less likely to be inhibition of any specific signaling pathway. The reduction in base SP-D expression with Ro 31–8220 was not significantly lower than media control. Further, while Gö-6976 significantly increased SP-D mRNA in HDE treated cells over HDE treatment alone, Ro 31–8220 did not. As a control for PKC inhibitor specificity, we measured both PKCα and PKCɛ activities in A549 cells pretreated for 1 hr with 1 μM Gö 6976 or 10 μM Ro 31–8220 (Fig 4b). PKCα was significantly inhibited by Gö 6976 (13.6 +/- 8.9 pmol/min/mg vs. media control of 84.3 +/- 3.1 pmol/min/mg; p<0.05), but not Ro 31–8220. Conversely, PKCɛ was significantly inhibited by Ro 31–8220 (15.1 +/- 3.6 pmol/min/mg vs. media control of 122.9 +/- 7.8 pmol/min/mg; p<0.05), but not by Gö 6976. This supports the application of these drugs to our assay.

### GPR116 in vitro and in a mouse model

**Fig 5:**
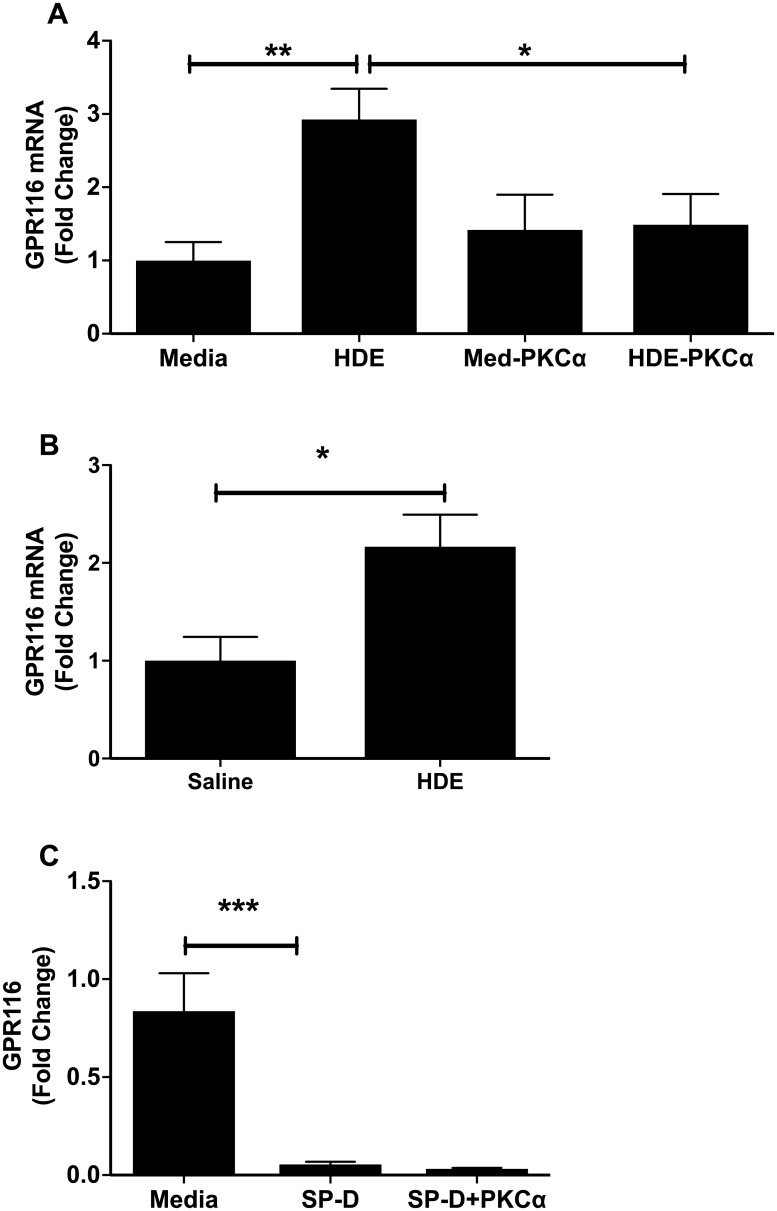
GPR116 is increased by HDE exposure. Treatment of A549 cells with HDE for 24 hr induced (a) a significant increase in GPR116 mRNA. Treatment of cells 1 hr prior HDE exposure with PKCα inhibitor Gö6976 was able to reverse this increase, but had no effect on unexposed cells. (b) mRNA from mice exposed to HDE for 24 hrs showed a similar increase. (c) Replacement of HDE with purified SP-D showed SP-D reduced GPR116 mRNA with no Gö6976 effect observed. Bar graphs represent averages with error bars denoting SEM (Cell cultures, N = 3 samples/group, repeated on three separate days. Mice, N = 4 mice/group). mRNA is given as fold change above media control levels. Statistical significance denoted by asterisks (*p < 0.05, **p < 0.01, ***p < 0.01) as compared to respective media treatment group.

Because others have shown that GPR116 can inhibit SP-D via a feedback loop [22], we looked to see if GPR116 levels are altered by dust treatment. We show that mRNA of GPR116 was elevated in response to HDE exposure in A549 cells (Fig 5a) and whole mouse lung (Fig 5b). Further, PKCα inhibitor abrogated the effects of HDE exposure, similar to what was seen with SP-D mRNA. PKCα inhibitor had no effect on its own on GPR116 mRNA expression, suggesting that HDE alters SP-D levels via a change in GPR116 expression. Because GPR116 is known to act as a feedback sensor for SP-D, we treated A549 with purified SP-D with or without PKC inhibitor (Fig 5c). SP-D significantly reduced GPR116 mRNA and was not sensitive to PKCα inhibitor, suggesting its feedback is a different mechanism from that seen with dust treatment.

## Discussion

Organic dust from CAFOs has been studied for a number of years. Much of the work focuses on innate immunity, and in particular the TLR pathways, such as TLR2 and TLR4. As these receptors have been shown to play roles in worker health, it has become apparent that other factors are likely to play critical roles in responses as LPS or peptidoglycan levels do not always predict outcomes [5]. This has led to examination of other innate mechanisms such as dust protease activity on protease activated receptors (PARs) [23], as well as co-exposures in workplaces, such as ammonia or CO_2_ [24].

The immune surfactants SP-A and SP-D have a broad range of ways in which they contribute to innate immunity, such as direct killing and neutralization actions, enhancement of phagocytosis, binding/inhibition of other innate immune receptors, or influencing intracellular signaling via receptor binding in both pro- or anti-inflammatory ways [11]. Both are expressed in high concentrations in the lung [25], and may play key roles in pathogen killing, innate immune regulation, and homeostasis [25]. We concentrated on SP-D because SP-A has two forms in humans, both with slightly different functionality [26], whereas in rodents, there is only one SP-A form. Further, distinguishing between subtypes by ELISA is potentially more of a challenge due to limited variation in SP-A forms.

First we examined HDE to determine if there was an effect on SP-A or SP-D in BAL from exposed mice. Both surfactants were significantly reduced in response to HDE (Fig 1a). Choosing to focus on SP-D we further confirmed that changes were also apparent in SP-D mRNA (Fig 1b). Given that mRNA levels were more sensitive we continued looking at SP-D mRNA.

Next, we examined a variety of settled dusts collected from agricultural operations such as grain elevators and CAFO operations, using the human bronchial epithelial cell line A549 (Fig 1c). We show here that organic dusts collected from these varied geographical and agricultural locations were all able to inhibit SP-D mRNA. Thus, this reduction in SP-D was not a response to the particulars of a single collection site, but a variety of sites with dusts that should show a large variation in their compositions. Further, changes in SP-D to HDE were present in a human lung cell line as well as the mouse model.

We continued to study this phenomenon in HDE from Nebraska as these samples have been well characterized for content [19]. Two of these components, LPS and peptidoglycan, are well known for their ability to bind key innate immune receptors, TLR4 and TLR2, respectively [27]. Both have also been implicated as major effectors of HDE inflammation [6]. Importantly, when we stimulated cells with these purified ligands, neither ligand altered levels of SP-D mRNA. Given the complexity of HDE and other organic dusts, other components may be responsible for the reductions we see. This is the subject of ongoing research. Interestingly, this component is heat labile, and once heated, HDE is still moderately stimulatory of SP-D, a situation we hope to study in the future.

We next determined if SP-D plays a role in the inflammatory response to HDE. We gave purified SP-D to A549 and PMA-differentiated THP-1 cells 1 hour prior to stimulation with HDE and looked for IL-8 expression in these cells. IL-8 is a key chemokine in induction of inflammation, migration of neutrophils to the lung, and commonly reported in HDE exposures [28]. A significant reduction in IL-8 was shown in SP-D pre-treated cells of both cell types, suggesting that SP-D levels could alter the level of inflammation in response to HDE, giving it further possible relevance to the inflammation we see in workers in response to HDE. This reduction in IL-8 could occur through a number of mechanisms, such as binding to targets to prevent attachment to innate immune receptors such as TLR4, CD14, or MD2 [12,29,30], binding of receptor ligands like LPS and peptidoglycan [31,32], or additional signaling through SIRP-1α [13]. Thus, inflammatory responses we see in workers may include reduction of potential inflammatory inhibitors such as SP-D.

Another common feature of HDE exposure is the sequential activation of PKCα followed by PKCɛ, which is responsible for the TNF-α-to-IL-8 cascade seen in such exposures [15–17]. Using the same PKC inhibition approach established in our previous studies, we examined if PKC is responsible for SP-D inhibition by HDE. Inhibition of PKCα, but not PKCɛ, was shown to reverse HDE inhibition of SP-D mRNA.

Thus, SP-D inhibition can be added to the established HDE cascade as another response requiring PKCα signaling. We hope to look for further downstream proteins involved in this pathway, but the finding of PKCα involvement helps to narrow possible candidates. Other likely targets for research can be defined as PKCα-induced cytokines such as IL-6 and IL-8 [16]. While PKCɛ treatment appeared to somewhat reduce background SP-D mRNA expression in media control treated cells, there was no apparent change in HDE treated SP-D mRNA levels.

Because GPR116 has been recently discovered to control the expression of SP-D [22,33], we next wanted to determine if HDE reduced SP-D through altered expression of GPR116, reasoning that increased receptor expression may reduce total SP-D levels, as there is evidence that SP-D is increased in mice with GPR116 knocked out [22]. HDE was able to increase the expression of GPR116 significantly (Fig 5a), both in the cell line and in mRNA from mouse lung (Fig 5b). When we examined a role for PKCα in this increase, we found that use of PKCα inhibitor brought GPR116 mRNA levels back down to control levels, suggesting that PKCα alters GPR116 levels in response to HDE, and not a downstream signal from GPR116 to mRNA expression. Interestingly, purified SP-D could reduce GPR116 expression, but this was not reversible by PKCα, suggesting that whatever mechanism that is involved with SP-D effects on GPR116, it is separate from that used by barn dust stimulation, and likely does not include PKCα.

Recently others have shown very convincingly the role of regulating guanine nucleotide-binding domain a, q, and 11 (Gq/11) in the inhibitory control of SP-D secretion [34]. GPR116 consists of two membrane bound, but non-covalently tethered peptides derive from proteolytic cleavage of GPR116 during synthesis [35]. The tethered c terminal fragment appears important in signaling as synthetic peptides mimicking it can induce G-protein receptor signaling that was blocked or inhibited by Gq/11 and phospholipase-c inhibitors [34,36], suggesting a role for both in the signaling pathway. We note that the pathway proposed for activity of Gq/11 via phospholipase C results in increased secondary messenger molecules such as diacylglycerol (DAG) production, a necessary component of PKCα activity (reviewed in [37]). This is a possible means of surfactant inhibition that appears to be in line with our work insofar that PKCα inhibition was able to block inhibition of mRNA for SP-D in the A549 cell line. The same however could be said of PKCɛ and DAG, so other factors are likely at work as well. In our study, this is not examined as a secretory control mechanism, but a possible change in SP-D protein synthesis. The end result, however, is control of SP-D. This is not to suggest that action of dust components has to be through GPR116, but that PKCα activation is a likely common feature of both systems. Multiple receptors and inflammatory pathways are involved in organic dust responses, several of which will lead to PKCα signaling as a crucial step [16].

Thus, we show that SP-D is significantly reduced in response to a component of organic dust both in human cell lines and in a mouse model system. PKCα is a critical part of this inhibitory signaling, both for SP-D and GPR116. There are obviously limitations to this work and more study of the pathway is required, both further confirming PKCα involvement as well as other potential signaling partners. Similarly, the specific component or components in these organic dusts that is responsible for SP-D inhibition still needs to be deduced. This work does, however, point to the possibility of reduced SP-D in animals and workers in CAFO environments, representing a potential compromise of one innate immune mechanism that may paradoxically result in an increase of inflammatory cytokines in the lung.
